# Radiomics’ Role in Predicting Distant Metastases, Recurrence and Survival Outcome in Rectal Cancer: A Systematic Review

**DOI:** 10.3390/cancers18091440

**Published:** 2026-04-30

**Authors:** Huda Mohammed, Hadeel Mohamed, Momoh Fofana, Samreen Jawaid, Mohamed Hersi, Omneya Alwani, Roshith Nair, Jayesh Sagar

**Affiliations:** 1 General Surgery Department, Craigavon Area Hospital, Craigavon BT63 5QQ, UK; huda.mohammed@southerntrust.hscni.net (H.M.); omneya.alwani@southerntrust.hscni.net (O.A.); roshithusha.nair@southerntrust.hscni.net (R.N.); 2 Faculty of Medicine, University of Khartoum, Khartoum 11115, Sudan; hadeelmohamed.404@gmail.com; 3 Emergency Department, Luton and Dunstable Hospital, Luton LU4 0DZ, UK; momoh.fofana@bedsft.nhs.uk; 4 College of Physician and Surgeon, 7th Central Street, DHA Phase-II, Karachi 75500, Pakistan; samreenjawaid@hotmail.com; 5 Internal Medicine Department, Luton and Dunstable Hospital, Luton LU4 0DZ, UK; mohamed.hersi4@nhs.net; 6 Surgery Department, Colorectal Surgery, Luton and Dunstable Hospital, Luton LU4 0DZ, UK

**Keywords:** rectal cancer, rectal carcinoma, radiomics, magnetic resonance imaging MRI, computed tomography CT, lymph node metastasis, distant metastasis, local recurrence, disease free survival, overall survival

## Abstract

Radiomics involves the extraction of quantitative information from medical images and can offer valuable insights into the management of rectal cancer (RC). We aim to systematically review the current literature on radiomics’ role in the prediction of lymph nodes and distance metastases, local recurrence and survival outcomes in rectal cancer. **Method:** This systematic review was conducted using the Preferred Reporting Items for Systematic Reviews and Meta-Analyses (PRISMA) guidelines and principles. We systematically searched PubMed, Cochrane, Google Scholar, and ResearchGate databases to identify the relevant articles. **Result:** After searching 3327 articles, only 50 articles were included after assessing the quality using a radiomics quality score (QRS) and finding a mean score of 14.74. Only eight studies used CT radiomics, with the rest based on MRI radiomics. Only three of the included studies are prospective, with the others being retrospective cohort studies and 14 having external validation. Most of the included studies concluded that radiomic models alone or combined models achieve better outcome predictions when compared with clinical and subjective analysis. **Conclusions:** Radiomics offers significant promise as a non-invasive tool for predicting lymph node status, distant metastasis (DM), recurrence, and survival outcomes in rectal cancer. There is a need for more robust prospective studies with cost benefit analysis for implementation into clinical practice.

## Simple Summary

Radiomics is a new imaging technique that uses computer analysis of scans such as MRI, CT, and PET to better understand rectal cancer without needing additional invasive tests. It can help predict important outcomes, including whether cancer has spread to lymph nodes or other organs, whether it may return, or how long patients might survive. In this review, we looked at studies using radiomics in the prediction of outcomes after treatment. Overall, these methods showed good ability to make predictions, often measured using accuracy scores such as AUC and C-index. However, the studies varied widely in how the images were processed and analyzed, making results difficult to compare. Many studies also had limitations, such as small sample sizes, retrospective designs, and lack of testing in independent patient groups. While the results are promising, more high-quality, standardized, and multicenter studies are needed to confirm its reliability and usefulness in everyday practice.

## 1. Introduction

Colorectal cancer (CRC) is the third most common malignancy worldwide and the second leading cause of cancer-related death [1]. The rectum accounts for 704,000 of the approximately 1.8 million CRC cases identified each year [2]. Rectal cancer continues to be a significant obstacle in oncology, necessitating ongoing research into novel treatment approaches [3]. Preoperative evaluations and forecasts, encompassing risk classification, therapeutic responses, long-term clinical outcomes, and genetic mutation status, are essential for optimizing individualized treatment methods [4]. Local recurrence and distant metastatic rates have decreased in recent decades due to the revision of multidisciplinary treatment approaches and the development of treatment techniques such as local excision, total mesorectal excision (TME), and neo-adjuvant chemoradiotherapy (nCRT) [5,6].

Lymph node metastasis significantly affects the choice of management options and the assessment of prognosis in rectal cancer. Research has demonstrated that patients with N2 stage have a worse chance of survival [7]. Thus, it is crucial for clinical diagnosis and treatment to ascertain whether lymph node metastases exist prior to treatment. However, predicting lymph node metastases is currently a significant difficulty in the diagnosis and treatment of rectal cancer, and judging based on lymph node morphological criteria is debatable. It is challenging to standardize the techniques and criteria for determining lymph node metastases because there are difficulties in matching the lymph nodes shown by preoperative imaging with those following surgery [8].

Imaging modalities such as CT and MRI possess limitations to ascertain lymph node (LN) status based on morphological criteria, as the characteristics of metastatic LNs with smaller short diameters frequently resemble those of nonmetastatic LNs [9,10]. Clinical practice has demonstrated that precise preoperative assessment can establish a dependable foundation for the selection of surgical techniques for RC, significantly enhancing prognosis and diminishing recurrence rates [8].

Quantitative imaging is gaining importance in clinical settings, offering clinicians valuable insights. This has led to increased interest in automated algorithms that extract and analyze complex data from images, known as radiomics [11,12]. Radiomics involves extraction of quantitative information from medical images using characterization methods, including texture, intensity-based statistics, and shape features [13,14]. It provides a comprehensive, multivariate, and thorough picture of a tumor’s phenotype, which is clearly difficult for even an experienced radiologist. However, the robustness and reproducibility of this type of technique are challenged as the extracted radiomic features depend on image quality, reconstruction, and processing decisions and settings, which naturally differ between institutions and operators [11]. In rectal cancer, radiomics has been applied for biological characteristic prediction, treatment efficacy evaluation, and therapeutic decision-making [15,16,17].

The aim of our study is to review the current literature on radiomics’ role in predicting lymph node metastases, distant metastases, local recurrence and survival outcomes in rectal cancer.

Although lymph node metastasis is considered an intermediate biomarker, it is closely linked to key clinical outcomes such as distant metastasis, recurrence, and survival, as all reflect tumor aggressiveness and progression within the metastatic cascade. These endpoints are interrelated in both biological behavior and clinical decision-making. Given that radiomics aims to capture the overall tumor phenotype, evaluating its role across both intermediate and final outcomes provides a more comprehensive assessment of its clinical utility in rectal cancer.

## 2. Radiomics

### 2.1. Radiomics Generally Includes the Following Steps

- Obtaining high-quality medical imaging data is a fundamental step for accuracy and for the repeatability of the radiomics model [18].
- Image segmentation is a crucial and challenging step in radiomics, as it involves defining the region of interest (ROI) or volume of interest (VOI). This ROI/VOI serves as the basis for subsequent feature extraction, making accurate segmentation essential for reliable results. It can be performed on an automatic, semi-automatic, or manual basis, with the latter being the standard method, although it is time-consuming and reader dependent [18,19,20,21].
- Feature extraction and selection are broadly categorized into four groups: size and shape features, first-order statistical features, second-order statistical features, and change-based features, including intensity, shape, texture, and wavelet features. The first-order features include mean, standard deviation, skewness, kurtosis, and entropy, while the second-order features are classified into a grey level co-occurrence matrix (GLCM), a run length matrix (RLM), and a grey level size zone matrix (GLSZM), which considers the spatial relationship between two pixels [18,19,22].
- Analyze (create models): Commonly utilized methods include Cox proportional hazard regression, logistic regression, least absolute shrinkage and selection operator (LASSO), random forest method, and support vector machine [18,23].
- Model implementation (classification and prediction): This is useful for tumor diagnosis, staging, prognosis, and therapy response prediction [18].

Large datasets with consistent imaging protocols are crucial for developing and validating radiomic models, as variations in protocols can affect textural features and reduce algorithm accuracy. To address this, using similar reconstruction algorithms or applying correction and calibration techniques can enhance dataset quality [14].

MRI-derived radiomic, artificial intelligence (AI) features, and quantitative imaging features, including diffusion kurtosis imaging (DKI), intra-voxel incoherent motion (IVIM), amide proton transfer weighted (APTw), which offer valuable insights into tumor function, microenvironment, and biological behavior [24,25,26,27].

Delta radiomics is a new radiomic concept referring to the modification of radiomics features by oncological treatments [28]. This approach seeks to develop predictive models by analyzing changes in radiomics features extracted from pre- and post-treatment images, incorporating individualized treatment response data. Various studies have explored the prediction of tumor behavior using clinical features alone or by combining texture analysis with morphological MRI and histopathological findings applied to either pre- or post-nCRT MRI, or both [29,30,31].

### 2.2. Magnetic Resonance Imaging (MRI)

Rectal high-resolution MRI (HRMRI) has become a key diagnostic tool for staging and accurately assessing factors influencing treatment and prognosis in rectal cancer, such as distance between the tumor to the mesenteric fascia, extramural vascular invasion (EMVI), lymph node presence, and peritoneal involvement [8]. According to some studies, vascular and lymphatic invasion are separate risk factors for lymph node metastases [32]. Lymph node metastases with a diameter of less than 5 mm are not usually detectable by MRI; therefore, several researchers have tried to predict lymph node metastasis indirectly using other markers that are partly accurate, like extramural vascular invasion [32,33]. According to Heijnen et al.’s study, the detection rate of diffusion-weighted imaging (DWI) for lymph nodes was almost 6% greater than that of traditional T2WI [34].

### 2.3. Computed Tomography (CT Scan)

Computed tomography (CT) is a commonly utilized radiological modality for clinical staging and therapeutic guidance; however, the absence of a standardized definition for lymph node metastasis (LNM) has compromised its diagnostic accuracy [35]. Contrast-enhanced CT (CE-CT) is also the predominant modality employed for the diagnosis of liver metastases (LM); however, its limited soft tissue resolution typically results in suboptimal sensitivity for detecting lesions measuring less than 1 cm in diameter [36,37]. Prompt identification of metachronous liver metastases (MLMs) facilitates timely interventions, including simultaneous resection or adjuvant treatments, and guides clinical decisions [38].

The role of CE-CT radiomics models, NCE-CT-based radiomics signature and dual energy CT have been reported in several studies as showing good performance in predicting lymph node metastasis (LNM) [39,40,41].

### 2.4. PET Scan

Fluorodeoxyglucose positron emission tomography combined with computed tomography (FDG-PET/CT) facilitates differentiation of post-treatment changes from pelvic recurrence, exhibiting superior sensitivity to CT for distant metastases and lymph node involvement [42]. PET/CT demonstrated a sensitivity of 75% for predicting pathological full response and 97% for detecting distant metastases [43]. However, PET/CT has important limitations as benign inflammation can mimic local recurrence and lead to false positives, whereas small lesions and tumors with low cellularity, such as mucinous and signet-ring cell carcinomas, may result in false negatives because of low 18F-FDG uptake [44,45,46,47,48,49]. Integrating pretreatment CT and PET radiomics features enhanced predictive accuracy to 88.1% in predicting treatment response [50]. A meta-analysis of 538 patients with locally advanced rectal cancer discovered that post-CRT PET/CT significantly reduced the maximal standardized uptake value in histopathologic responders [51].

PET/MRI appears more accurate for T staging, with similar N and M staging performance to PET/CT, owing to the superior soft-tissue contrast of MRI [52,53].

### 2.5. Methods and Study Design

**Figure 1:**
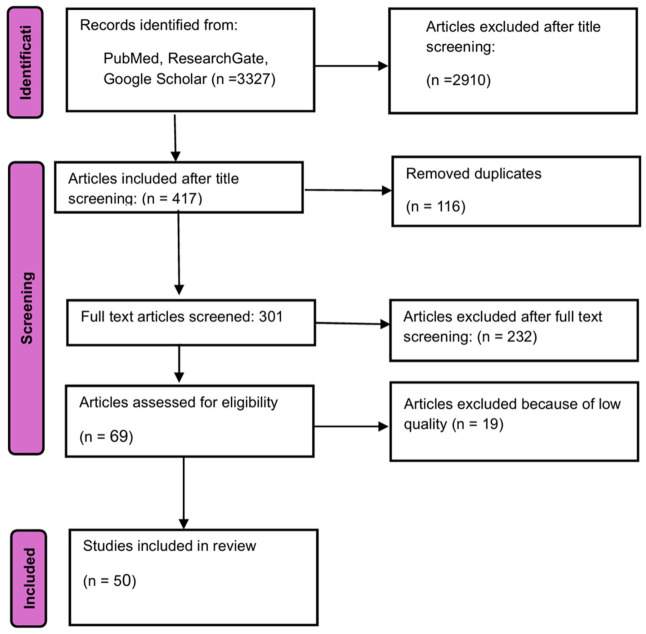
PRISMA chart.

This systematic review was conducted using the Preferred Reporting Items for Systematic Reviews and Meta-Analyses (PRISMA) guidelines and principles for studies published until 30 June 2025. We systematically searched the PubMed, Cochrane, Google Scholar, and ResearchGate databases. Keywords, including (“rectal cancer” OR “rectal carcinoma”) AND (“radiomics”) AND (“distant metastasis” OR “recurrence”) AND (“MRI” OR “CT” OR “PET” OR “magnetic resonance imaging” OR “computed tomography”), were used to identify the articles. Two independent researchers reviewed all the articles identified. The list of relevant articles was reviewed systematically to identify related studies. All articles were evaluated using inclusion and exclusion criteria. A PRISMA flow diagram was used to illustrate the study selection process (Figure 1) [54].

The results of included studies were summarized in tables, including study characteristics such as author, year, study design and imaging-related variables such as imaging modality (e.g., MRI, CT, PET/CT), segmentation method (manual, semi-automatic, or automatic), methods of feature selection, number of selected features, and validation. Due to heterogeneity in imaging protocols, feature extraction methods, and outcome measures, a narrative synthesis was conducted to qualitatively integrate the findings across studies. Where appropriate, results were grouped according to clinical endpoints, including lymph node metastasis, distant metastasis, recurrence, and survival outcomes. The results of the included studies were presented using performance metrics such as the area under the curve (AUC) and concordance index (C-index).

### 2.6. Inclusion and Exclusion Criteria

#### 2.6.1. Inclusion Criteria

1. Population: Adult patients (≥18 years) with histologically confirmed rectal carcinoma, at any disease stage.
2. Intervention: Radiomics from medical imaging (MRI, CT, PET/CT) to predict lymph node metastasis, distant metastasis, local recurrence, or disease-free/overall survival.
3. Comparison: Histopathological confirmation, clinical follow-up, or established imaging-based criteria.
4. Outcomes: Quantitative assessment of model performance for predicting lymph node metastasis, distant metastasis, local recurrence, disease-free survival (DFS), or overall survival (OS).
5. Study design: Original retrospective or prospective studies, cohort, or diagnostic accuracy studies.
6. Language and accessibility: English, full-text articles in peer-reviewed journals.

#### 2.6.2. Exclusion Criteria

1. Population: Non-rectal colorectal cancers, animal studies, in vitro or phantom studies.
2. Intervention: Non-radiomics studies, studies focusing on treatment response or tumor staging without recurrence/metastasis assessment.
3. Outcomes: No performance metrics, no recurrence, metastasis, or survival endpoints.
4. Study design: Reviews, meta-analyses, editorials, letters, conference abstracts, and case reports of overlapping patient cohorts.
5. Technical limitations: Insufficient methodological detail, non-peer-reviewed preprints.

### 2.7. Quality Assessment of the Studies

**Figure 2:**
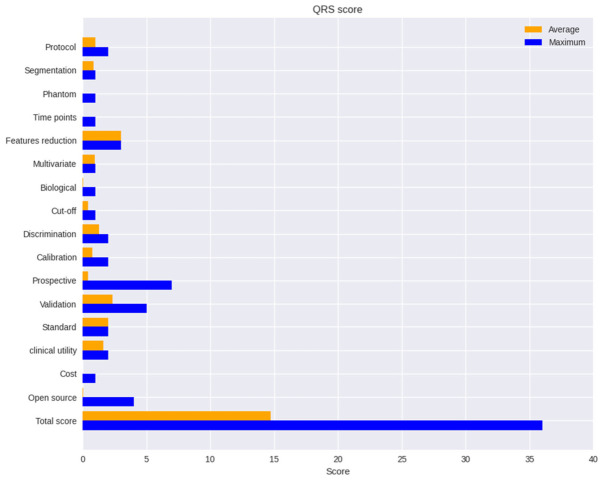
Average QRS scores of the included studies.

We systematically evaluated the included studies for quality using a standardized quality check tool for radiomic studies called a radiomic quality score (RQS) [55]. Figure 2 shows average QRS scores of the included studies. The QRS range from −8 to 36 points. Our included studies’ quality total scores ranged from 11 to 23 and are shown in diagram number 1. Quality Assessment of Diagnostic Accuracy Studies (QUADAS-2) was used for the assessment of risk of bias and applicability [56]. The assessment of the included studies is summarized in Supplementary Table S1.

## 3. Result

A total of 3327 articles were identified in the database. After the title screening, 417 articles were included. Following full-text screening, 69 articles were found to be eligible. QRS was used for quality screening, and the final number of papers included in this review, after the exclusion of 19 papers due to their low quality [8,36,57,58,59,60,61,62,63,64,65,66,67,68,69,70,71,72,73], was 50 [36,39,41,74,75,76,77,78,79,80,81,82,83,84,85,86,87,88,89,90,91,92,93,94,95,96,97,98,99,100,101,102,103,104,105,106,107,108,109,110,111,112,113,114,115,116,117,118,119]. The total number of patients included in all studies is 12,284. The majority of the papers include MRI scans and only nine include CT scans [36,41,43,81,115,116,117,118,119]. Twenty-four MRI studies are multiparametric [29,41,74,75,76,77,78,79,80,81,82,83,84,85,86,87,88,89,90,91,92,93,94,95,96,97,98,99,100,101,102,103,104,105,106,107,108,109,110,111,112,113,114]. Only three of the included studies are prospective [104,106,110], with the others being retrospective cohort studies and 14 having external validation [29,75,77,78,81,82,86,87,88,94,95,103,106,115].

Six studies are delta radiomic, while the remaining images are just pretreatment images. In all studies, image segmentation was done manually, except for two that were performed automatically and three that were done semi-automatically. The predominantly utilized feature selection methods are least absolute shrinkage and selection operator (LASSO), COX regression analysis, and minimum redundancy maximum relevance (mRMR). The final number of [29,41,74,75,76,77,78,79,80,81,82,83,84,85,86,87,88,89,90,91,92,93,94,95,96,97,98,99,100,101,102,103,104,105,106,107,108,109,110,111,112,113,114] selected features in the research ranges from 2 to 65 features. The characteristics of the included studies are listed in Table 1. The results presented in the tables were obtained using C-index and AUC for model discrimination.

Lymph node prediction was included in 26 trials. Most of these studies indicate that radiomic and combined clinical–radiomic models have better performance than the clinical model. Table 2 shows studies involved in the prediction of LNM.

Five studies investigated the recurrence rate and recurrence-free survival rate which are shown in Table 3.

Ten research developed radiomics models to predict distant metastases in rectal cancer. Table 4 shows the studies included.

Table 5 shows the four studies that examined overall survival.

Eleven articles investigated disease-free survival, two of which focused on DM-free survival, as summarized in Table 6.

## 4. Discussion

In this systematic review, we aimed to assess the ability of radiomics to predict recurrence, lymph nodes and distant metastasis, disease-free survival and overall survival in LARC. We included fifty MRI and CT studies in this review and our findings indicate that radiomic models demonstrated good performance in predicting outcomes in patients with LARC.

Clinical models based on clinicopathological factors and MRI semantics features have limitations in capturing tumor heterogeneity, potentially leading to variable prognoses among patients with similar characteristics [103]. In contrast, radiomics features extracted from mpMRI of the entire tumor provide a comprehensive quantification of intratumor heterogeneity and an established prognostic factor [103].

Established prognostic indicators of adverse survival outcomes in RC include MRI-detected positive circumferential resection margins (CRMs), extramural venous invasion (EMVI), tumor deposits, and lateral pelvic lymph node (LPLN) enlargement [120,121,122]. Zhao et al. have developed a multi-sequence MRI-based radiomic nomogram (T1WI, T2WI, and proton density) that achieved an AUC of 0.899 for EMVI detection, exceeding radiologists’ accuracy [123].

MRI enables accurate clinical staging of T-stage and distance from anatomical margins, thereby determining the benefit of preoperative radiotherapy based on the estimated risk of LR [72]. Given the correspondence between pathological T-stage and current MRI staging, the nomograms can be utilized as a supplementary tool to identify patients for whom preoperative radiotherapy can be safely omitted [72].

Suspected LR at the anastomotic site is challenging to distinguish just with standard MRI, as it is intricately associated with the presence of staplers in anastomotic areas, fibrotic scar tissue, treatment-related alterations, and inflammatory lesions. These phenomena may be interpreted by subjective and nonquantitative aspects in pictures, resulting in a lack of clinical importance [124]. The MRI-based radiomics model showed efficacy as a non-invasive method for the early detection of local recurrence in RC cases with clinically suspected lesions at the anastomotic location [124,125].

Several researchers have suggested that DWI and DCE-MRI could be useful in distinguishing locally recurring lesions from benign tissue, especially in perirectal soft tissue and the presacral fascia [126,127,128].

The delta-radiomics signatures from 2- (2D) and 3-dimensional (3D) features extracted from T2WI can promptly predict local recurrence after chemoradiotherapy (CCRT) and surgery in LARC, as concluded by Jeon s et al. [83]. Furthermore, combining radiomic features with the clinical predictors outperforms the clinical model in the prediction of LR [78].

A study conducted by Niu et al. demonstrated that the MRI radiomics model exhibited a significantly superior AUC compared with the CT model, with respective AUCs of 0.785 and 0.721 in the validation cohort, indicating that MRI-based radiomics is more accurate at assessing N staging [41]. This is due to the high soft tissue resolution and multiparametric features of MRI, which improve tissue information and enable a more thorough tumor characterization. In contrast, CT is restricted to displaying density differences between tissues [129,130,131].

Qu, W et al.’s study was the first research in the evaluation of non-enlarged LNM in rectal cancer to utilize DWI-based radiomics and to compare both DWI images and ADC maps [74]. The study’s findings reveal that radiomic features generated from ADC maps (rADC model) perform better at predicting non-enlarged LNM status in rectal cancer than features taken from DWI pictures (rDWIb800, cDWIb800), cADC, and T2WI. Moreover, the rADC-based radiomics technique outperformed radiologists’ subjective assessments [74].

Despite potential of LPLN dissection to prevent local recurrence and enhance survival, its application is hindered by technical difficulties, postoperative complications, and adverse effects on sexual and urinary function. This can be prevented by selective use of LPLN dissection, ensuring it is employed in conjunction with TME and only when imaging indicates enlarged LPLNs [115,132,133,134].

A study has reported that a radiomics model (AUC = 0.842) outperformed radiologists (AUCs = 0.734, 0.668) in identifying metastatic lateral pelvic lymph nodes (LPLNs), suggesting enhanced discriminative ability. Combining radiomics with expert interpretation can improve treatment planning for rectal cancer patients, enabling more accurate LPLN dissection or nCRT selection and potentially improving outcomes [127]. Several studies have demonstrated that the radiomic model outperformed the subjective assessment by radiologist [77,85,87,95].

Challenges in establishing accurate correlation between histopathology nodes and MRI-identified nodes may explain the reliance on radiomic signatures from the primary tumor to predict extranodal extension (ENE), according to O’Sullivan et al. [114]. Additionally, small lymph nodes (<5 mm) with positive ENE are particularly problematic, as their size hinders accurate delineation on MRI, making it difficult for radiologists to define reliable ROI [114]. Furthermore, radiomic evaluation of intratumoral regions on T2-weighted imaging revealed a predictive capacity for ENE, with an AUC value of 0.842, as demonstrated in recent research [110].

According to Niu et al., the NCE-CT-based radiomics signature shows greater efficacy in lymph node metastasis (LNM) prediction in rectal cancer when compared with CE-CT [39]. Notably, combining NCE-CT and CE-CT radiomics models improve prediction performance. Furthermore, integrating CT and MRI radiomics models yields superior predictive efficacy, outperforming other radiomics models [39].

Dual-energy CT (DECT) is an emerging imaging technology that enables the selective quantification of different image materials [40]. A study found that a radiomics model based on 120kVp-like images showed a higher performance in predicting LNM in rectal cancer patients, outperforming, with an AUC of 0.922, both CT morphological indicators and DECT parameters. However, using an iodine map did not improve the performance, suggesting that this approach is a valuable tool for predicting LNM in rectal cancer patients [41].

In a study conducted by Liu et al. to evaluate the role of pre-nCRT MRI radiomics characteristics in the prediction of synchronous distant metastasis (SDM), pre-nCRT MRI radiomics showed good performance, with an AUC of 0.827 [99]. The study demonstrated that integrating radiomics characteristics into the clinical model increased predictive performance for SDM in rectal cancer patients, producing a better AUC of 0.827 vs. 0.779 and offering excellent sensitivity, specificity, positive predictive value, and negative predictive value in the validation cohort [99].

Utilizing a support vector machine, a retrospective study by Liang et al. identified MRI radiomics features that differentiated metastatic from non-metastatic patients, discovering predictors of metachronous liver metastasis with an AUC of 0.87 in a cohort of 108 patients [119]. Another study conducted by Liu M et al. demonstrated that the integration of a radiomics nomogram with tumor markers surpassed the predictive capability of the radiomics signature alone, achieving an improved AUC of 0.944 (vs. 0.866) and exhibiting enhanced sensitivity in the validation cohort for predicting SLM in RC patients [100].

Previous research has demonstrated the utility of MRI radiomics in predicting MLM in rectal cancer, yielding promising results [78,119]. Notably, these studies have primarily focused on intratumoral regions, potentially overlooking valuable information in the peritumoral area [103]. More recently, Li et al. analyzed 130 rectal cancer patients, constructing radiomics models using T2WI and DWI sequences [78]. The fusion model’s AUC in the test set was 0.911 (95% CI: 0.825, 0.978), outperforming previous studies [78].

Pre-treatment radiotherapy planning CT images can successfully predict the survival outcomes in LARC and the features from both primary tumor and mesorectum are useful in the prediction [117]. In addition, combining radiomic features with clinical and pathological predictors can significantly improve the prediction performance, as demonstrated in the study by Chuanji et al., which investigated multi-parametric MRI radiomic models [107]. Additionally, other studies have confirmed the superiority of the combined model in the prediction of overall survival [84,116].

Radiomics models have demonstrated good predictive ability for DFS, successfully stratifying patients into high-risk and low-risk groups, which suggests the potential for guiding personalized treatment [103].

A T2WI-based radiomic model achieved good performance when predicting DFS after nCRT in the Lui J et al. study [113]. Additionally, this study identified three matching signatures to predict DFS in LARC patients by extracting radiomic characteristics from pre- and post-therapy MRI scans. In order to predict DFS, it also assessed the predictive parameters of the pre-, post-, and delta-radiomic signatures. With a C-index of 0.724, their results showed that the pre-nCRT radiomic signature performed better [113]. The quality and dependability of the data extracted from the pre-nCRT MRI images, which offered more detailed insights into the unique features of the tumor, may have contributed to this improved performance [113].

MRI-detected EMVI and clinical–radiomic nomogram-based LNM identified as independent the adverse predictors of 3-year RFS in multivariate analysis, consistent with the established correlation between MRI-reported EMVI and distant recurrence [120]. A prognostic model incorporating these indicators demonstrated robust performance in predicting 3-year RFS [106].

Across the included studies, radiomic and combined models consistently demonstrated superior discriminatory performance compared with clinical models alone, with combined approaches yielding the highest AUCs across endpoints such as lymph-node metastasis, distant metastasis, recurrence, DFS, and OS. This pattern reinforces the additive value of radiomic features when integrated with conventional clinical variables. However, interpretation of these findings is limited by the substantial heterogeneity observed across studies. The cohorts varied widely in sample size, demographics, disease stage, and treatment pathways, all of which influence model stability and generalizability. Imaging protocols differed markedly between studies, including multiple MRI sequences, CT techniques, and multiparametric combinations, introducing variability in feature extraction and limiting cross-study comparability. Moreover, the prediction tasks themselves were heterogeneous, with studies targeting different clinical endpoints of varying biological complexity, which naturally produces a wide range of AUC values. Methodological diversity, including differences in feature-selection strategies, model structures, and the number of radiomic features used, also contributes to inconsistent performance estimates, particularly in smaller datasets prone to overfitting. Finally, the predominance of internal validation and the scarcity of external validation across studies likely inflate reported performance and restrict the applicability of these models to broader clinical settings. In conclusion, while the overall trend supports the promise of radiomics, the heterogeneity across studies underscores the need for methodological standardization and rigorous external validation to ensure reliable, reproducible, and clinically translatable results.

### Limitations and Challenges

The implementation of radiomics in clinical practice is hindered by several challenges, including variability in imaging protocols and scanners, inconsistencies in methodologies, and issues related to feature extraction and unbalanced data, which can lead to inflated model performance. Internal and external validation is essential to ensure model accuracy; however, data-sharing initiatives are often limited by patient privacy concerns.

## 5. Conclusions

Radiomics offers significant promise as a non-invasive tool for predicting distant metastasis, recurrence, and survival outcomes in rectal cancer, with many studies demonstrating encouraging diagnostic and prognostic performance. However, the current studies remain limited by methodological heterogeneity, small single-center cohorts, inconsistent reporting, and a lack of robust external validation. Although there is methodological heterogeneity across the studies, it seems that most of the included studies showed that the combined radiomic and clinical models outperform the traditional assessment tools in predicting lymph node involvement, distant metastasis, recurrence, and survival outcomes in rectal cancer. There is a need for robust studies with the characteristics of large sample size, standardized imaging protocols, consistent methodologies, involvement of external validation cohorts, and transparent reporting adhering to standardized reporting guidelines so as to ultimately strengthen radiomics research and clinical translation.

## Figures and Tables

**Table 1:** Characteristics of the studies included in the review.

Author, Year	Study Design	Imaging Modalities	Segmentation	Features Selection Method	Number of Features	External Validation
Qu W et al. [74], 2025	Retrospective	T2WI, rDWI, and cDWI, rADC, cADC	Manual	LASSO	4	No
Fu S et al. [75], 2025	Retrospective	T2WI/DWI MRI	Manual	LASSO + Cox regression	25	Yes
Meng Y et al. [76], 2024	Retrospective	T2WI and ADC images	Manual	LASSO	21	No
Ma S et al. [77], 2023	Retrospective	T1WI/T2WI/DWI/HR-T2WI MRI	Automatic	LASSO	5	Yes
Li Z et al. [78], 2025	Retrospective	T2WI, DWI, T1WI, and CET1WI	Manual	LASSO	8	Yes
Xie F et al. [79], 2022	Retrospective	T1WI, T2WI CE-T1WI	Manual	LASSO + Cox regression	11	No
Zhao R et al. [80], 2025	Retrospective	T2W, ADC	Manual	LASSO + COX	7	No
Feng Y et al. [81], 2025	Retrospective	CE-CT/T2WI/DWI	Semi-automatic	LASSO	13	Yes
Niu Y et al. [39], 2023	Retrospective	NCE-CT, T2WI, CE-T1WI/DWI MRI	Manual	Recursive feature elimination	CT15/MRI 17	No
Yoo J et al. [82], 2025	Retrospective	T1WI/T2WI	Semi-automatic	LASSO	6	Yes
Jeon S et al. [83], 2019	Retrospective	T2W/DWI	Manual	LASSO	22	NO
Nie K et al. [84], 2022	Retrospective	DCE-MRI/T1WI/T2WI/DWI	Manual	LASS0 + COX regression	8	No
Ye Y et al. [85], 2024	Retrospective	T1WI/T2WI MRI	Manual	LASSO regression algorithm	13 t1, 10 T2, combined18	No
Wei Q et al. [86], 2024	Retrospective	T2WI/DWI MRI	Manual	LASSO	13	Yes
Ao W et al. [87], 2025	Retrospective	T1WI/T2WI/DWI	Automatic	MRMR + LASSO	30	Yes
Liu Z et al. [88], 2020	Retrospective	T2WI/DWI	Manual	LASSO + MRMR	4	Yes
Meng X et al. [89], 2019	Retrospective	T1W, DWI, DCE, T2W	Manual	LASSO + MRMR	10	No
Cui Y et al. [90], 2022	Retrospective	T2WI, DKI, and CE-T1WI	Manual	Feature stability and Cox proportional hazards model	6	No
Li C et al. [91], 2021	Retrospective	(T2WI) and ADC image	Manual	LASSO	13	No
Zhou X et al. [92], 2020	Retrospective	T1WI, T2WI, DWI, CE-T1W MRI	Manual	LASSO	13	No
Zhao Q et al. [93], 2024	Retrospective	T1WI, T2WI, DWI	Manual	LASSO	11	No
Yao X et al. [94], 2024	Retrospective	T2WI, DWI, and CE-T1WI	Manual	MRMR + LASSO	26	Yes
Zheng Y et al. [95], 2024	Retrospective	T2WI, DWI, CE-T1W MRI	Manual	MRMR + LASSO	29	Yes
Fang Z et al. [96], 2023	Retrospective	T2WI, ADC MRI	Manual	Correlation analysis, recursive feature elimination with logistic regression classifier (RFE-LR)	2	No
Yang Y et al. [97], 2021	Retrospective	HR-T2WI	Manual	LASSO + MRMR	9	
Dong X et al. [98], 2023	Retrospective	T2WI	Manual	MRMR + SVM-RFE	14	No
Liu H et al. [99], 2019	Retrospective	T2WI	Manual	Random forest algorithm	6	No
Liu M et al. [100], 2020	Retrospective	T2WI	Manual	LASSO	5	No
Song G et al. [101], 2022	Retrospective	HR-T2WI-MRI	Manual	LASSO	45, 62, 65, and 35,	No
Yan H [102], 2024	Retrospective	T2WI-based MRI	Manual	LASSO	4	No
Shi S et al. [103], 2025	Retrospective	T2WI-based MRI	Manual	LASSO	19	Yes
Li J et al. [104], 2020	Prospective	T2WI	Manual	Multi-objective optimization with the iterative multi-objective immune algorithm (IMIA	10	No
Ma J et al. [105], 2024	Retrospective	T2WI	Manual	LASSO + Cox regression	5	No
Li H et al. [106], 2023	Prospective	T2WI	Manual	LASSO regression algorithm	10	Yes
Chuanji Z et al. [107], 2022	Retrospective	T2WI	Manual	LASSO	10	No
Wang C et al. [108], 2023	Retrospective	T2WI	Manual	LASSO + cox	15	No
Qin S et al. [109], 2024	Retrospective	T2WI	Manual	LASSO	46	No
Sun Y et al. [29], 2024	Retrospective	T2WI	Manual	LASSO	43	Yes
Li H et al. [110], 2024	Prospective	T2WI	Manual	LASSO		No
Li Y et al. [111], 2023	Retrospective	DWI	Manual	LASSO logistic regression analysis	8	No
Tibermacine H et al. [112], 2021	Retrospective	T2-weighted MRI	Manual	Recursive feature elimination and extra tree classifier	9	No
Liu J et al. [113], 2024	Retrospective	HR- T2WI	Manual	Pearson correlation analysis and ANOVA or relief	12	No
O’Sullivan et al. [114], 2025	Retrospective	T2WI MRI	Manual	LASSO	6	No
Nakanishi R et al. [115], 2020	Retrospective	PVP-CT.	Manual	LASSO logistic regression	9	Yes
Wang F et al. [116], 2022	Retrospective	CE-CT	Manual	LASSO + COX	2	
Wang D et al. [41], 2022	Retrospective	Dual energy CT scan	Manual	LASSO	10 M/8	No
Wang J et al. [117], 2019	Retrospective	NCE- CT	Manual	Cox regression	21	No
Yuan H et al. [118], 2022	Retrospective	Triphasic CT	Manual	GBDT	55	No
Liang M et al. [36], 2022	Retrospective	PVP CE-CT	Manual	LASSO + MRMR	16	No
Li M et al. [119], 2020	Retrospective	CE-CT	Manual	LASSO	3	No

**Table 2:** Lymph nodes prediction.

Author	Clinical		Radiomic		Combined		
	Training	Validation	Training	Validation	Training	Validation	Comment
Yan H et al. [102]	0.729	0.655	0.841	0.835	0.843	0.833	Combined model demonstrated good performance in the prediction of LN metastasis and was even superior to the clinical model.
Meng Y et al. [76]	0.68	0.60	0.87	0.70	0.89	0.71	Radiomic model showed good prediction ability for LN metastasis for T3 rectal cancer with a *p* value of 0.047 compared with the clinical model in the validation group.
Ma S et al. [77]	0.662	0.64/0.734	0.903	0.735/0.802 **	0.921	0.908/0.884 **	The nomogram exhibits better performance than radiomic and radiologist evaluation, with a *p* value of 0.01 when comparing radiomics and subjective evaluation.
Song G et al. [101]	-	0.671	0.83	0.82	-	-	Radiomic models with four methods of segmentation achieved good diagnostic performance.
Nakanishi R et al. [115]	0.83	0.80	0.91	0.90	0.83	0.94	Radiomic score showed good discrimination of lateral pelvic LN after CRT, even better than the clinical model, with a *p* value of 0.0298.
Qu W et al. [74]	-	-	0.859	0.911	-	-	Radiomic model based on rADC achieved better performance than subjective assessment in predicting non enlarged LN metastasis with *p* value of 0.008.
Dong X et al. [98]	0.62	0.59	-	-	0.74	0.62	Combined model performance is almost equivalent to clinical model.
Niu Y et al. [39]	0.699	0.657	0.888/0.848 ✦	0.721/0.785 ✦	0.947	0.828	The MRI radiomic model demonstrated better performance than the CT model with the clinical–radiomic model outperforming all models.
Yang Y et al. [97]	0.76	0.68	0.83	0.80	0.86	0.83	The nomogram achieved good predictive performance and even better performance than radiomics with *p* value of 0.003 and clinical model with *p* value of 0.017 in the training group.
Yoo J et al. [82]	-	-	0.819	0.772/0.821 *	0.83	0.762/0.829 *	Radiomics model achieved good predictive performance in predicting metastatic LPLN similar to combined model.
Wang D et al. [41]	-	0.755	0.915	0.922	-	-	Radiomic signature based on CT demonstrated better performance than other clinical–radiological predictors with *p* value of 0.0473
Li J et al. [104]	0.73	-	0.92	-	0.94	-	Combined models have good predictive performance.
Ye Y et al. [85]	0.82	0.80	0.89	0.86	0.92	0.91	Radiomics model achieved good performance and when combined with subjective evaluation it performed best with a *p* value of 0.001.
Li H et al. [106]	0.67	0.64	0.77	0.76	0.84	0.78	Good predictive performance of clinical–radiomics nomogram, with a similar performance to the clinical model in the external validation.
Wei Q et al. [86]	0.687	0.623	0.978	0.831	-	-	MR radiomic before and after nCRT achieved good prediction of LNM after nCRT in LARC.
Ao W et al. [87]	0.78	0.79	0.97	0.99	0.98	0.99	The predictive performance of the DLRS and nomogram models was superior to that of the physician model with *p* value of <0.001.
Qin S et al. [86]	-	-	0.933	0.826	-	-	Combined model achieved a better performance.
Sun Y et al. [29]	0.80	0.574/0.571	0.942	0.741/0.713 *	0.996	0.690/0.726 *	Radiomics model based on the largest short axis lymph node achieved reliable performance in predicting lateral pelvic LNM.
Yuan H et al. [109]	-	-	0.626	0.627	0.656	0.638	The clinical–radiomic nomogram achieved good performance in predicting the LNM status of RC.
Li H et al. [110]	0.736	0.667	0.707	0.667	0.799	0.723	The nomogram of multi-regional radiomics achieved better performance in the validation group rather than in the training group.
Li Y et al. [111]	0.81	0.849	0.781	0.827	0.856	0.88	The combined model showed good predictive performance for the preoperative prediction of LNM and higher accuracy.
Meng X et al. [89]	-	-	0.752	0.677	0.804	0.697	The radiomic model achieved similar predictive performance with the subjective radiologist MRI assessment.
Li C et al. [91]	0.811	0.781	0.875	0.822	0.937	0.884	The radiomics model and clinical model achieved comparable performance in the prediction of LNM with better performance when combined in nomogram.
Zhou X et al. [92]	0.696	0.701	0.787	0.783	0.826	0.818	Combined model demonstrated good performance in the prediction of LNM after neoadjuvant therapy, especially post-therapeutic MRI T1-2 tumors.
Zheng Y et al. [95]	0.763	0.742	0.787	0.797	0.837	0.834	Combined has good performance, although clinical and radiomics have similar performance.
Fang Z et al. [96]	0.807	0.753	0.824	0.834	0.913	0.912	Clinical–delta ADC radiomic model achieved good predictive performance in predicting LNM and can predict 5-year DFS in early rectal cancer.

* External validation ** two external validation ✦ CT/MRI result.

**Table 3:** Recurrence rate prediction.

Author	Clinical		Radiomics		Combined		
	Training	Validation	Training	Validation	Training	Validation	Comment
Li Z et al. [78]	0.696	0.694/0.685 *	0.786	0.780/0.793 *	0.825	0.812/0.831 *	Combined model showed better performance in predicting early recurrence in LARC compared with clinical (*p* 0.001 in training and 0.004 in validation) and radiomic model (*p* value of 0.005 in training and 0.048 in validation)
Wang F et al. [116]	0.68 ± 0.04	-	0.63 ± 0.05	-	0.72 ± 0.04	-	The combined model outperforms the clinical model in predicting local recurrence in RC with *p* value of 0.02
Jeon S et al. [83]	-	-	0.951 ± 0.026	-	-	-	Delta radiomic signatures successfully predicted the outcomes and were independent prognostic factors.
Li H et al. [106]	-	-	-	-	0.748	0.706	clinical-radiomics nomogram achieved good performance for predicting 3 years recurrence free survival
O’Sullivan et al. [114]	-	-	-	0.75	-	-	Radiomics signatures showed good performance in predicting recurrence in patients with locally advanced rectal cancer

* External validation.

**Table 4:** Prediction of DM.

Author	Clinical		Radiomics		Combined		
	Training	Validation	Training	Validation	Training	Validation	
Shi S et al. [103]	0.713	0.705	0.926	0.864	0.959	0.925	The nomogram combining conventional radiomics, habitat model and CA19-9 exhibited the best predictive performance across all cohorts.
Liu H et al. [99]	0.792	0.779	-	-	0.847	0.827	Combined model could achieve a better performance in predicting SDM in rectal cancer.
Liu M et al. [100]	-	-	0.836	0.866	0.918	0.944	Radiomics nomogram combined with tumor markers was superior to the radiomics signature alone, with high predictive performance for SLM in RC patients.
Jeon S et al. [83]	-	-	0.894 ± 0.048	-	-	-	Delta radiomic signatures successfully predicted the outcomes and were independent prognostic factors.
Feng Y et al. [81]	0.77	0.67/0.55 *	0.85	0.75/0.73 *	0.91	0.79/0.73 *	Multi-modal radiomics model integrating from both primary tumors and pre-metastatic liver regions achieved superior predictive performance for LM in RC.
Ma J et al. [105]	0.766	0.801	0.697	0.619	0.842	0.805	Combined radiomics with CEA, CA199, and KRAS mutation demonstrated better performance in predicting liver metastasis compared with clinical models
Li M et al. [119]	0.782	0.766	0.709	0.687	0.842	0.802	Clinical–radiologic risk model that showed better prognostic performance than rad score for predicting distant metastasis of RC within 3 years of surgery, with a *p* value of 0.020 for the validation cohort.
Liang M et al. [36]	0.65	0.58	0.84	0.84	0.85	0.85	Radiomics model based on preoperative whole-liver PVP CE-CT showed better performance in predicting MLM within 24 months after RC surgery. The combined model and radiomics showed similar performance.
Zhao Q et al. [93]	0.825	0.819	-	-	0.839	0.868	Combined model has better prediction of 3-year DM risk compared with radiomic, with a *p* value of 0.048 in validation and 0.013 in all cohorts.
Yao X et al. [94]	0.82	0.82	0.83	0.85	0.92	0.89	Combined model showed higher performance than the other models in the prediction of DM.

* External validation.

**Table 5:** Prediction of OS.

Author	Clinical		Radiomics		Combined		
	Training	Validation	Training	Validation	Training	Validation	Comment
Wang F et al. [116]	0.64 ± 0.06	-	0.62 ± 0.09	-	0.66 ± 0.06		The combined model has higher performance to predict the OS compared with the clinical model, with a *p* value of 0.03.
Nie K et al. [84]	0.66	0.56	0.88	0.89	0.91	0.91	Survival prediction in stage II and III rectal cancer is best achieved by the combined model, with a *p* value <0.05 when compared with TNM staging and clinical model.
Chuanji Z et al. [107]	0.768	0.72	0.809	0.847	0.872	0.944	Comprehensive model integrating imaging, clinicopathological risk and radiomic model achieved better performance in prediction of 5-year overall survival than any single model, with a *p* value of <0.05.
Wang J et al. [117]	0.713	0.672	0.675	0.655	0.745	0.73	The radiomic model can improve the prediction performance of the clinical model when combined with the prediction of overall survival in LARC, with a *p* value of 0.044.

**Table 6:** Prediction of DFS and DMFS.

Author	Clinical		Radiomics		Combined		
	Training	Validation	Training	Validation	Training	Validation	
Fu S et al. [75]	0.65	0.55	0.728	0.72	0.775	0.739	Combined radiomic model achieved better performance compared with clinical model in the assessment of DFS, with a *p* value of 0.001 in training and validation.
Xie F et al. [79]	0.578	0.611	0.937	0.73	0.942	0.752	Combined post-treatment models with postoperative pathological factors outperformed the pretreatment models for predicting the 3-year progression-free survival of LARC with a *p* value of <0.05.
Zhao R et al. [80]			0.811	0.848	0.95	0.872	Nomogram is an effective tool to identify high-risk patients with DM and develop personalized treatment.
Wang F et al. [116]	0.64 ± 0.11	-	0.57 ± 0.12	-	0.66 ± 0.12	-	The combined model has better performance compared with the clinical model, with a *p* value of 0.04 in DMFS and 0.20 in DFS prediction.
Jeon S et al. [83]	-	-	0.897 ± 0.046	-	-	-	Delta radiomic signatures successfully predicted the outcomes and were independent prognostic factors.
Wang C et al. [108]	-	-	0.72	0.69	0.78	0.8	Pathological–radiomic nomogram has superior performance over the conventional pathological staging system in predicting DMFS.
Wang J et al. [117]	0.678	0.658	-	-	0.683	0.643	Radiomics features, when combined with clinical features, provided a model with similar predictive performance in progression-free survival.
Liu Z et al. [88]	0.682	0.595	0.847	0.809	0.855	0.848	Combined model achieved good performance in the initial diagnosis stage with improved performance when incorporating postoperative pathologic indicators in the prediction of DFS in patients with LARC.
Cui Y et al. [90]	0.661/0.696 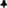	0.721/0.747 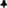	0.713	0.708			Radiomics model with pre- and postoperative features improved performance in the prediction of DFS compared with clinical models.
Tibermacine H et al. [112]	0.71 ± 0.06/0.63 ± 0.07	0.66 ± 0.19/0.75 ± 0.13	0.89 ± 0.09/0.77 ± 0.09	0.74 ± 0.10/0.73 ± 0.06	-	-	Radiomics models can predict DFS in patients with locally advanced rectal cancer, outperforming clinical models in training groups but comparable in the validation group.
Liu J et al. [113]	0.802	0.755	0.835	0.724	0.924	0.833	Combined pre- and post-therapy radiomic models improve prediction of DFS compared with clinical models alone, with a *p* value of 0.001.

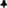
 Pre- and post-radiomic models.

## Data Availability

No new data were created or analyzed in this study.

## Supplementary Materials

The following supporting information can be downloaded at https://www.mdpi.com/article/10.3390/cancers18091440/s1, Table S1: Risk of bias assessment of the included studies using QUADAS-2.

## Abbreviations

ADC	Apparent diffusion coefficient
AI	Artificial intelligence
APTw	Amide proton transfer-weighted imaging
AUC	Area under the curve
b-value	Diffusion-weighting factor in DWI
cADC	Conventional apparent diffusion coefficient
cDWIb800	Conventional diffusion-weighted imaging at b = 800 s/mm^2^
CE-CT	Contrast-enhanced computed tomography
CI	Confidence interval
C-index	Concordance index
COX	Cox proportional hazards regression
CRC	Colorectal cancer
CRM	Circumferential resection margin
CRT	Chemoradiotherapy
CT	Computed tomography
cT3/4	Clinical tumor stage 3 or 4
DCE-MRI	Dynamic contrast-enhanced magnetic resonance imaging
DECT	Dual-energy computed tomography
DFS	Disease-free survival
DKI	Diffusion kurtosis imaging
DM	Distant metastasis
DWI	Diffusion-weighted imaging
EMVI	Extramural venous invasion
ENE	Extranodal extension
ESGAR	European Society of Gastrointestinal and Abdominal Radiology
FDG	Fluorodeoxyglucose
18F-FDG	Fluorine-18 fluorodeoxyglucose
FDG-PET/CT	Fluorodeoxyglucose positron emission tomography/computed tomography
GLCM	Gray-level co-occurrence matrix
GLSZM	Gray-level size Zone matrix
HRMRI	High-resolution magnetic resonance imaging
IVIM	Intravoxel incoherent motion
Ktrans	Volume transfer constant (DCE perfusion parameter)
kVp	Kilovoltage peak
LARC	Locally advanced rectal cancer
LASSO	Least absolute shrinkage and selection operator
LM	Liver metastasis
LN	Lymph node
LNM	Lymph node metastasis
LPLN	Lateral pelvic lymph node
LPLND	Lateral pelvic lymph node dissection
LR	Local recurrence
MLM	Metachronous liver metastasis
mpMRI	Multiparametric magnetic resonance imaging
MRI	Magnetic resonance imaging
MRgRT	Magnetic resonance-guided radiotherapy
M staging	Metastasis staging (TNM classification)
NELNM	Non-enlarged lymph node metastasis
NPV	Negative predictive value
N staging	Nodal staging (TNM classification)
OS	Overall survival
pCR	Pathological complete response
PET	Positron emission tomography
pN2	Pathological nodal stage 2
PPV	Positive predictive value
PRISMA	Preferred Reporting Items for Systematic Reviews and Meta-Analyses
QUADAS-2	Quality assessment of diagnostic accuracy studies
RC	Rectal cancer
rADC	Radiomics-based apparent diffusion coefficient
rDWIb800	Radiomics-based diffusion-weighted imaging at b = 800 s/mm^2^
RFS	Recurrence-free survival
RLM	Run length matrix
ROI	Region of interest
ROC	Receiver operating characteristic
RQS	Radiomics quality score
SDM	Synchronous distant metastasis
SLM	Synchronous liver metastasis
SVM	Support vector machine
TME	Total mesorectal excision
TNM	Tumor–node–metastasis staging system
T1WI	T1-weighted imaging
T2WI	T2-weighted imaging
T staging	Tumor staging (TNM classification)
