# Supplementary material for: Radiomics’ Role in Predicting Distant Metastases, Recurrence and Survival Outcome in Rectal Cancer: A Systematic Review
Source: Cancers (Basel). 2026 Apr 30;18(9):1440. doi: 10.3390/cancers18091440 (PMC13163067; doi:10.3390/cancers18091440)
Supplement: Supplementary file 1 [file cancers-18-01440-s001.zip › Table S1.pdf]

Table S1: Risk of bias assessment of the included studies using QUADAS-2

No	Author, year	Risk of Bias				Applicability concern		
		Patient selection	Index Test	Reference standard	Flow and timing	Patient selection	Index Test	Reference standard
**1**	Qu W et al. 2025	Low	Low	Low	Low	Low	Low	Low
**2**	Fu S et al. 2025	Low	Unclear	Low	Low	Low	Low	Low
**3**	Meng Y et al. 2024	Low	Low	Low	Low	Low	Low	Low
**4**	Ma S et al. 2023	Low	Low	Low	Low	Low	Low	Low
**5**	Li Z et al. 2025	Low	Low	Low	Low	Low	Low	Low
**6**	Xie F et al. 2022	Low	Unclear	Low	Low	Low	Low	Low
**7**	Zhao R et al. 2025	Low	Low	Low	Low	Low	Low	Low
**8**	Feng Y et al. 2025	Unclear	Low	Low	Low	Low	Low	Low
**9**	Niu Y et al. 2023	Low	Low	Low	Low	Low	Low	Low
**10**	Yoo J et al. 2025	Low	Low	Low	Low	Low	Low	Low

**11**	Jeon S et al. 2019	Unclear	High	Low	Low	Low	Low	Low
**12**	Nie K et al. 2022	Low	Unclear	Low	Low	Low	Low	Low
**13**	Ye Y et al. 2024	Low	Unclear	Low	Low	Low	Low	Low
**14**	Wei Q et al. 2024	Low	Low	Low	Low	Low	Low	Low
**15**	Ao W et al. 2025	Low	Low	Low	Low	Low	Low	Low
**16**	Liu Z et al. 2020	Low	Unclear	Low	Low	Low	Low	Low
**17**	Meng X et al. 2019	Low	Unclear	Low	Low	Low	Low	Low
**18**	Cui Y et al. 2022	Low	Low	Low	Low	Low	Low	Low
**19**	Li C et al. 2021	Low	Low	Low	Low	Low	Low	Low
**20**	Zhou X et al. 2020	Low	Low	Low	Low	Low	Low	Low
**21**	Zhao Q et al. 2024	Low	Low	Low	Low	Low	Low	Low
**22**	Yao X et al. 2024	Low	Low	Low	Low	Low	Low	Low
**23**	Zheng Y et al. 2024	Low	Low	Low	Low	Low	Low	Low

**24**	Fang Z et al. 2023	Low	Low	Low	Low	Low	Low	Low
**25**	Yang Y et al. 2021	Low	Unclear	Low	Low	Low	Low	Low
**26**	Dong X et al. 2023	Low	Low	Low	Low	Low	Low	Low
**27**	Liu H et al. 2019	Low	Low	Low	Low	Low	Low	Low
**28**	Liu M et al. 2020	Low	Unclear	Low	Low	Low	Low	Low
**29**	Song G et al. 2022	Low	Low	Low	Low	Low	Low	Low
**30**	Yan H 2024	Low	Unclear	Low	Low	Low	Low	Low
**31**	Shi S et al. 2025	Low	Low	Low	Low	Low	Low	Low
**32**	Li J et al. 2020	Low	Low	Low	Low	Low	Low	Low
**33**	Ma J et al. 2024	Low	Low	Low	Low	Low	Low	Low
**34**	Li H et al. 2023	Low	Low	Low	Low	Low	Low	Low
**35**	Chuanji Z et al. 2022	Low	Low	Low	Low	Low	Low	Low
**36**	Wang C et al. 2023	Low	Unclear	Low	Low	Low	Low	Low

**37**	Qin S et al. 2024	Low	Low	Low	Low	Low	Low	Low
**38**	Sun Y et al. 2024	Low	Unclear	Low	Low	Low	Low	Low
**39**	Li H et al. 2024	Low	Low	Low	Low	Low	Low	Low
**40**	Li Y et al. 2023	Low	Unclear	Low	Low	Low	Low	Low
**41**	Tibermacine H et al. 2021	Low	Low	Low	Low	Low	Low	Low
**42**	Liu J et al. 2024	Low	Low	Low	Low	Low	Low	Low
**43**	O'Sullivan et al. 2025	Low	Unclear	Low	Low	Low	Low	Low
**44**	Nakanishi R et al. 2020	Low	Low	Low	Low	Low	Low	Low
**45**	Wang F et al. 2022	Low	Low	Low	Low	Low	Low	Low
**46**	Wang D et al. 2022	Low	Unclear	Low	Low	Low	Low	Low
**47**	Wang J et al. 2019	Low	Low	Low	Low	Low	Low	Low
**48**	Yuan H et al. 2022	Low	Low	Low	Low	Low	Low	Low
**49**	Liang M et al. 2022	Low	Low	Low	Low	Low	Low	Low

**50**	Li M et al. 2020	Low	Low	Low	Low	Low	Low	Low
